# Optimizing Telework with Exercise: An Exploration of the Curvilinear Relationship between Telework Intensity and Work Engagement

**DOI:** 10.3390/bs14080674

**Published:** 2024-08-03

**Authors:** Weiwei Huo, Bingqian Liang, Yongmei Liang, Mengli Song, Yixin Wang

**Affiliations:** SILC Business School, Shanghai University, Shanghai 200444, China; huoweiwei@shu.edu.cn (W.H.); liangbingqian003@shu.edu.cn (B.L.); songmengli@shu.edu.cn (M.S.); wyx2091@shu.edu.cn (Y.W.)

**Keywords:** telework intensity, self-control, physical exercise, work engagement

## Abstract

The sudden COVID-19 crisis disrupted people’s pace of life. Meanwhile, telework has gradually attracted public attention and become a regular mechanism for organizations. In this context, self-regulation theory was utilized to explore the impact of telework intensity on work engagement. Data were collected from 304 employees across three time points, and the results supported a curvilinear relationship between telework intensity and work engagement. Specifically, it was discovered that employees exhibited the highest levels of work engagement at moderate levels of telework intensity. Additionally, based on the strength model of self-control, the research identified self-control as playing a mediating role between telework intensity and work engagement. Finally, the study revealed that the duration of physical exercise moderated the relationship between telework intensity and self-control. Long-term physical exercise was found to prolong and enhance the positive effects of telework intensity on self-control, thereby boosting employee work engagement. This study provided unique and valuable insights into maintaining employee engagement in telework.

## 1. Introduction

Given the uncertainty of the environment and the diverse office needs of employees, telework has always been an auxiliary work mode within organizations [1]. A global telework report for 2023 revealed that, from April to May 2023, full-time employees spanning 34 countries worked from home an average of 0.9 days per week [2]. Moreover, organizations have observed tangible benefits. 65% of Chief Financial Officers reported that telework directly reduced office space, cutting facility costs by more than 10% annually in a 2022 study by IWG (International Workplace Group). Telework will continue to be adopted by organizations in future work models, and this trend has significantly accelerated and is widely accepted. However, telework presents a critical difference from traditional office work, namely the reduction in external supervision [3].

With the lack of external supervision in telework, management has concerns about employee work engagement [4]. Work engagement is marked by vigor, dedication, and absorption, with engaged employees exhibiting passion and proactive involvement [5,6]. The debate on telework’s impact on work engagement is ongoing. Some research underscores its positive effects, arguing that telework can foster an environment conducive to higher work engagement [7,8,9]. However, others note that the absence of supervision in telework can lead to distractions from household and social activities, potentially reducing enthusiasm and work engagement [10,11].

Cultivating work engagement is crucial for achieving both organizational efficiency and employee well-being [10,11]. However, past research on telework’s impact on work engagement has shown mixed results [7,8,9,10,11,12,13]. Therefore, this paper focuses on exploring the effects of telework on work engagement, considering both its benefits and potential risks.

**Figure 1:**
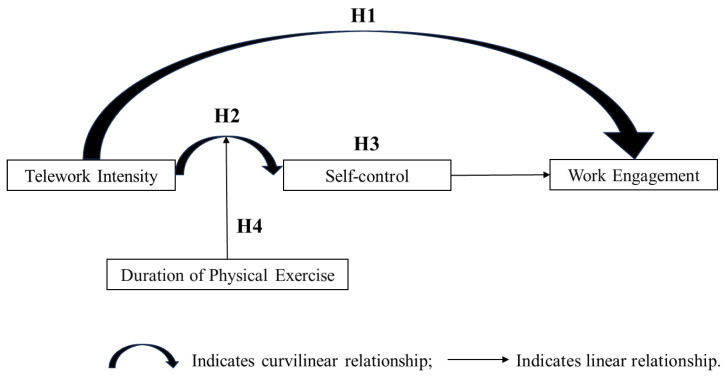
Theoretical model.

We propose that the relationship between teleworking and employee work engagement is not merely linear but exhibits a curvilinear effect due to the varying intensities of telework. The self-regulation theory (SRT) highlights that self-regulation operates through three key processes: standards, monitoring, and motivation [12]. These processes are impacted differently across various levels of telework intensity, positively and negatively affecting work engagement. Furthermore, self-control is a potential mediator in the curvilinear relationship between telework intensity and work engagement. According to SRT, individuals tend to self-control, aligning themselves with environmental demands [14]. In telework settings lacking external oversight, employees are inclined to self-regulate, suggesting an initial increase in self-control [15,16,17]. However, the strength model of self-control also notes that the prolonged depletion of self-control resources can lead to failures in subsequent self-regulatory behaviors [18,19], indicating that self-control evolves with changes in telework intensity. This evolution of self-control, affecting physical, cognitive, and emotional aspects, influences work engagement [20,21]. Moreover, considering that telework extends beyond traditional workplace settings, we explore factors associated with non-traditional work environments. Physical exercise, characterized by its intensity, frequency, and duration, aims to enhance physical health [22,23]. We believe that the duration of physical exercise can modulate the relationship between telework intensity and self-control by improving cognitive functions and mental health. Figure 1 depicts our theoretical model.

Our research makes notable contributions in three areas. Firstly, we explore the impact of telework on work engagement. Scholars have long sought to unravel the paradoxical nature of telework [4,24,25]. However, previous studies often treated telework as a binary variable, categorizing individuals as teleworkers or non-teleworkers [26,27]. Grounded in the SRT, our study investigates the intensity of telework and its effects on work engagement. This helps to identify the levels of telework intensity that can maintain employees’ work engagement, moving beyond the simplistic debate of whether organizations should adopt telework. Secondly, from the perspective of internal self-perception, we explore the potential mediating mechanism between telework intensity and work engagement. While previous studies have primarily focused on external factors such as work–family conflict [28,29,30] to examine the impacts of telework, this paper offers new insights into the effects of telework on work engagement from the perspective of self-control. This contributes to a better understanding of how self-control or other deep-seated traits may influence employee behavior and outcomes. Lastly, our research enriches the study of telework from a physiological health perspective. Given that long-term physical exercise can counteract and replenish the depletion of self-control resources, we explore the moderating role of the duration of physical exercise in the relationship between telework intensity and self-control. This has innovative and unique implications for optimizing telework strategies from a physiological health standpoint and for promoting employee engagement, bridging the fields of physical health and work psychology. Furthermore, by incorporating the applicability of physiological health factors into the SRT, our study broadens the application of the theory.

### 1.1. The Relationship between Telework Intensity and Work Engagement

SRT posits that individuals possess the capacity for self-regulation, enabling them to adjust and control their attitudes and behaviors in response to changes in the external environment [12,14]. This self-regulation mechanism operates through three critical subprocesses: standards, monitoring, and motivation [12].

Telework typically refers to working outside traditional workplaces using remote communication tools or computer-based technology [31]. Telework intensity is based on how much time telework takes in a week; the more time it takes, the higher the intensity. When telework intensity ranges from low to moderate, it can serve as a supportive organizational condition, fostering increased flexibility and improved work–life balance [26]. Moreover, telework gives employees greater autonomy and control [32]. These positive factors enhance self-regulation motivation, helping to maintain commitment and enthusiasm for work through self-regulation in telework [33]. Additionally, the boost in self-regulation motivation strengthens employees’ intrinsic motivation, highlighting the intrinsic value and meaning of their work [34]. Such intrinsic motivation is crucial to work engagement [5]. When telework intensity is low to moderate, typically not exceeding three days a week, it may not significantly impact the standards and monitoring required for self-regulation mechanisms [31,35,36]. However, initial levels of telework can act as a supplementary condition, enhancing employees’ self-regulation motivation and triggering positive psychological states and intrinsic motivation, thereby improving work engagement.

As telework intensity rises, employees immersed in telework for long stretches may increasingly encounter vague, uncertain, and conflicting organizational standards, complicating their self-regulation efforts. Successful self-regulation hinges on clear standards [12,37] and monitoring [12,38]. Typically, employees would align their behavior with these standards to self-adjust and meet objectives. However, the extended duration of telework often comes with a lack of explicit standards and external oversight, leaving employees uncertain about when to make necessary adjustments. Thus, this prolonged lack of clarity and supervision obstructs effective self-regulation in high telework intensity environments. Employee confusion and insecurity can lead to reduced work engagement [39]. Furthermore, the difficulty in setting and sticking to goals may weaken their sense of purpose at work, further eroding work engagement [40]. Thus, we propose the following hypothesis:

**Hypothesis** **1.** *Telework intensity has an inverted-U-shaped relationship with work engagement such that a moderate level of telework intensity results in the highest level of work engagement.*

### 1.2. The Relationship between Telework Intensity and Self-Control

Self-control is the intentional adjustment of actions to achieve long-term goals, showing one’s ability to resist short-term temptations based on inner traits [41,42]. SRT deals with extensive self-regulation mechanisms, treating self-control as an active regulatory behavior. The strength model of self-control offers a more detailed theory about self-control, conceptualizing self-control as a specific type of resource [18,19,38].

SRT and the strength model of self-control provide a clear explanation for telework scenarios. It shows why self-control levels often rise when telework intensity moves from low to moderate. Specifically, telework allows employees to perform tasks outside the main office, exposing them to more distractions and temptations [43]. SRT suggests that individuals strive to control their emotions and resist impulses that could worsen their situation, aligning themselves with environmental demands. Thus, in telework, employees actively engage in more specific self-control processes to better focus on their work. Spending extra time practicing self-control can significantly boost self-control levels [44]. In essence, in the early stages of telework, employees take the initiative to exercise self-control, and self-control ability gradually increases after training.

However, the strength model of self-control also indicates that self-control resources are limited. With the relentless depletion of these resources, employees can reach a state of “ego depletion”, leading to failures in later self-control actions [18,19]. When telework intensity moves from moderate to high, the long-term depletion of self-control resources starts to weaken self-control. Moreover, in anticipating ongoing telework and the need for self-control, employees might save resources for future self-control demands, thereby reducing the amount of self-control resources allocated to activities, such as engaging less in work or taking brief breaks [45]. Thus, as telework intensity rises from moderate to high, levels of self-control begin to drop.

In summary, self-control improves as telework intensity increases from low to moderate levels. However, as intensity rises from moderate to high, self-control diminishes. Thus, we propose the following hypothesis:

**Hypothesis** **2.** *Telework intensity has an inverted-U-shaped relationship with self-control, such that a moderate level of telework intensity results in the highest level of self-control.*

### 1.3. The Mediating Role of Self-Control

SRT suggests that individuals can manage their emotions and resist impulses to meet environmental demands. Telework often lacks external supervision [4], which means that self-control will play a crucial role in telework. Initially, employees consciously apply self-control to meet telework demands, so self-control strengthens when telework intensity increases from low to moderate. However, the strength model also cautions about the limited nature of self-control resources. Sustained exertion can lead to “ego depletion”, impairing subsequent self-control efforts [18,19]. As telework intensity increases from moderate to high, the continuous consumption of self-control resources can weaken self-control. Essentially, moderate telework can improve self-control, but high telework intensity can diminish it.

Kahn [46] was the first to introduce the concept of employee engagement, suggesting that engaged employees are physically, cognitively, and emotionally involved in their work roles, experiencing a sense of meaningfulness, psychological safety, and availability. Employee engagement often reflects an individual’s dedication to their work, manifesting as a positive psychological state characterized by focus, energy, and commitment [5]. Firstly, enhanced self-control can increase their physical engagement in work. This is because individuals with high self-control tend to proactively set goals and protect themselves from temptations or distractions, making them more focused on completing work tasks than those with lower self-control [20]. Moreover, employees with solid self-control capabilities are better equipped to handle work stress and challenges and face difficulties and challenges more confidently, thus exhibiting higher work zeal and positivity [21]. Simultaneously, as employees with higher levels of self-control are more effective at managing stress and coping with challenges, lower stress levels contribute to a stronger sense of psychological safety, as employees feel safer and less threatened in the workplace. Therefore, enhancing employees’ self-control capabilities can also increase their cognitive and emotional investment in their work. It is evident that improving employees’ self-control can enhance their work engagement on physical, cognitive, and emotional levels, indicating a positive correlation between self-control and work engagement.

Previous studies on telework have supported the notion of self-control as a mediating variable. For instance, research has examined the mediating role of self-control between leadership behaviors and work–life balance in telework [47]. It indicates that self-control is an important intermediary factor affecting employees’ behavior and performance in telework. In this study, we propose that the relationship between telework intensity and work engagement is partially mediated by self-control, which is supported by three main effects in the theoretical section: (a) an inverted-U-shaped relationship between telework intensity and work engagement (i.e., Hypothesis 1); (b) an inverted-U-shaped relationship between telework intensity and self-control (i.e., Hypothesis 2); (c) a linear relationship between self-control and work engagement. Some studies have employed similar logic to explain the mediating role in this curvilinear relationship [48,49]. In summary, varying telework intensity affect employees’ self-control, with moderate telework intensity potentially enhancing self-control, which, in turn, can improve work engagement. Therefore, we propose the following hypothesis:

**Hypothesis** **3.** *Self-control partially mediates the relationship between telework and work engagement.*

### 1.4. The Moderating Role of Physical Exercise

Physical exercise, characterized by its intensity, frequency, and duration, is a sports activity that enhances physical health [22,23]. We propose that the duration of physical exercise can moderate the relationship between telework intensity and self-control by promoting cognitive functions and mental health. Long-term physical exercise typically involves engaging in physical activities or sports over an extended period, indicating that individuals continuously participate in regular physical training rather than sporadically. On one hand, long-term physical exercise can specifically boost an individual’s endurance and adaptability to the mental fatigue associated with sustained cognitive tasks and enhance attention and concentration as well as overall cognitive functions [50,51]. This enhancement in endurance can mitigate the depletion of self-control resources [51], thus delaying the consumption of self-control resources brought about by high telework intensity and extending the positive impact of telework on self-control as much as possible. Consequently, with long-term physical exercise, the inflection point of the inverted-U curve between telework intensity and self-control shifts to the right.

On the other hand, long-term physical exercise can aid in replenishing self-control resources. Numerous studies have found that physical exercise among employees can effectively reduce work-related stress, lower feelings of anxiety and depression, and stimulate positive emotional experiences [52]. Exercise is known to have antidepressant effects on the body [53]. After exercising, an increase in heart rate and blood circulation is observed. Other beneficial effects of exercise include changes in cortisol levels, steroids, endorphins, and cytokines, which influence an individual’s mood [54]. These changes are also considered to be pathophysiological causes of affective disorders. Additionally, some reports suggest that exercise can alter neuroactive substances in the central nervous system [55]. A positive psychological state for a long time is crucial in replenishing self-control resources [56,57]. When people maintain a positive psychological state over a long period, they can effectively manage the stress and challenges associated with high-intensity telework, reducing the depletion of self-control resources. Moreover, individuals with a positive mindset usually have better emotional regulation abilities. They can recover more quickly from the negative emotions that may arise from high-intensity telework, preventing the excessive consumption of self-control resources. Therefore, long-term physical exercise can enhance the positive curve effect of telework on self-control. Consequently, under the influence of long-term physical exercise, the inflection point of the inverted-U curve between telework intensity and self-control moves upwards.

In contrast, short-term physical exercise does not have the same effect on resource recovery and endurance enhancement as long-term physical exercise. The accumulation of resources and endurance enhancement are often the result of long-term cumulative effects. Short-term physical exercise struggles to produce these cumulative effects on both physiological and psychological levels, especially for self-control resources that require time to build and strengthen gradually. Moreover, from a psychological perspective, the formation of habits, improvement in emotional stability, and development of a positive mindset require time [58]. Short-term physical exercise may not provide sufficient opportunities to foster the development of these psychological adaptations, limiting its contribution to self-control resources. Thus, we propose the following hypothesis:

**Hypothesis** **4.** *The duration of physical exercise moderates the curvilinear relationship between telework intensity and self-control, such that an upper-right inflection point occurs on the inverted-U shape for individuals with a long (vs. short) duration of physical exercise.*

## 2. Methods

### 2.1. Participants and Procedure

This study uses a questionnaire survey to collect data. To avoid common methodological bias, this study used a longitudinal paired questionnaire survey at three time points for data collection: Time point 1 (December 2022), which measures employees’ basic information and telework intensity; Time point 2 (February 2023), which mainly measures self-control and the duration of physical exercise; Time point 3 (April 2023), which mainly measures employee engagement. Repeated measures were taken from subjects who participated in the study at three different time points. Participants were identified using the last four digits of their phone numbers to track their responses across the three time points. By collecting data at multiple time points, we aimed to mitigate common method bias and to obtain more reliable and valid results. Multiple time points help in separating the measurement of different variables, reducing the influence of memory effects and consistency motives and lowering the common method variance [59]. We collected 347 pieces of data in T1, 325 pieces of data in T2, and 304 pieces of data in T3. The experimental mortality was 6.34% from T1 to T2, and the experimental mortality was 6.46% from T2 to T3. After eliminating noncompliant time-point matches and filling out the required questionnaires, 304 valid questionnaires were ultimately obtained. The effective response rate of the questionnaire was 87.6%. In the sample of effective employees, males accounted for 51.3% and females accounted for 48.7% in terms of gender. In terms of age, 67.8% were aged 35 and below; in terms of educational background, 88.1% of them were undergraduate or above. Employees mainly came from the consulting services industry (30.3%), while the majority of employee positions were enterprise managers (46.4%) and ordinary employees (33.2%).

### 2.2. Measures

The measurements of self-control and work engagement were translated from English into Chinese following recommended back-translation procedures [60]. The items were measured on a 5-point Likert scale ranging from 1 (“strongly disagree”) to 5 (“strongly agree”). We provide an overview of all the items in Appendix A. 

#### 2.2.1. Telework Intensity

We referred to Virick et al.’s study (2010) [61] to measure telework intensity by asking participants to indicate the average number of days a week spent working from home. The options ranged from 0.5 days to 7 days.

#### 2.2.2. Self-Control

Self-control was measured using a thirteen-item scale developed by Tangney et al. [62]. A sample item is “I can resist temptation very well”. The α reliability for this scale was 0.91.

#### 2.2.3. The Duration of Physical Exercise

The duration of physical exercise was measured using a one-item scale. The item is “How long can you persist in exercising (exercise such as yoga, running, aerobics, cycling, etc.)”. Options included 0–3 months, 3–6 months, 6–12 months, 1–2 years, and two years and above.

#### 2.2.4. Work Engagement

Work engagement was measured using a nine-item scale developed by Schaufeli et al. [63]. The α reliability for this scale was 0.92.

#### 2.2.5. Control Variables

We controlled for gender because studies have found that female employees tend to exhibit higher levels of engagement compared with male employees [64]. Regarding age, research by Chaudhary and Rangnekar (2017) indicates significant differences in engagement across age groups [65], with younger employees showing lower levels of engagement compared with older employees. The reason cited is that younger employees, particularly those under the age of 25, are more likely to change jobs and are motivated by opportunities for growth and development rather than stability. Additionally, Garg (2014) found a negative correlation between educational qualification and employee engagement in a study across various industries in India [66]. Furthermore, numerous studies on work engagement have been conducted across different industries and professions, suggesting that varying work contexts may influence the fluctuations in work engagement [67,68].

### 2.3. Data Analysis

Our theoretical model does not involve latent variables or complex measurement models. To assess the direct and indirect relationships among observed variables, we utilized path analysis in SPSS 23.0 to test the hypothesized relationships.

A curvilinear relationship:

Y = b_0_ + b_1_ × X + b_2_ × X^2^ + e (1)

The inflection point is calculated as

−b_1/_(2 × b_2_) (2)

In our theoretical development about the moderating role of exercise, we do not expect the duration of exercise to change the concave versus convex nature of the curvilinear relationship. Because we are concerned with whether high levels of fitness will prolong the benefits of telework intensity on self-control (i.e., shifts in the inverted-U-shape’s inflection points), we followed the recommendations of Pierce and Aguinis [59] (p. 326, Equation (4)) and tested the moderated curvilinear relationship using the following equation:

Y = b_0_ + b_1_ × X + b_2_ × M + b_3_ × XM + b_4_ × X^2^ + e (3)

where X is the predictor, M is the moderator, and Y is the outcome variable.

In this model, when the moderator is at high versus low levels (M_H_ and M_L_), the inflection points are

−(b_1_ + b_3_ × M_H_)/(2 × b_4_) (4)

−(b_1_ + b_3_ × M_L_)/(2 × b_4_) (5)

The shift in the inflection points is the difference between these two values. Previous studies [48,69,70] used the same approach to test moderated curvilinear effects. Calculating the shifts in the inflection points and the instantaneous indirect effects requires examining non-normally distributed compound coefficients. We followed the Monte Carlo bootstrapping approach recommended by Preacher and Selig [71] to create 95% Monte Carlo bootstrapped confidence intervals (CIs, with 20,000 resamples).

## 3. Results

### 3.1. Preliminary Analysis

Table 1 presents the descriptive statistics and correlations among the study variables. Education is negatively correlated with work engagement (r = −0.12, *p* < 0.05), age is negatively correlated with work engagement (r = −0.13, *p* < 0.05), telework intensity is positively correlated with work engagement (r = 0.12, *p* < 0.05), the duration of physical exercise is positively correlated with work engagement (r = 0.28, *p* < 0.01), and self-control is positively correlated with work engagement (r = 0.71, *p* < 0.05). As previously shown, the reliability coefficients of the research scale are all above 0.90, indicating good internal consistency reliability of the scale. Before testing the hypotheses, confirmatory factor analysis was conducted using Lisrel 8.7 to test the discriminant validity and measurement parameters of the two variables. The two-factor model fit the data reasonably well (*χ^2^* = 461.654, comparative fit index [CFI] = 0.93, Tucker–Lewis index [TLI] = 0.92, root mean square error of approximation [RMSEA] = 0.063) and significantly better than the single-factor model (∆*χ*^2^ = 395.52, *p* < 0.001, CFI = 0.82, TLI = 0.81, RMSEA = 0.10). The results suggest good discriminant validity of the research variables. In addition to the procedural techniques adopted in the data collection, statistical remedies were also employed to test the CMV. Specifically, we examined the CMV using Harman single-factor analysis, following procedures described by Podsakoff et al. [72]. We found that two factors were extracted, and the variance contribution rate of the first factor extracted was 46.291% (<50%). Therefore, the CMV did not significantly impact the model. In addition, for possible common method deviations in this study, confirmatory factor analysis was used to test the common method deviation of all self-assessment items, and the results showed that the model fit was poor, with *χ*^2^/*df* = 4.10, CFI = 0.82, TLI = 0.81, RMSEA = 0.10, suggesting that there is no significant common method deviation issue.

### 3.2. Hypotheses Testing

**Figure 2:**
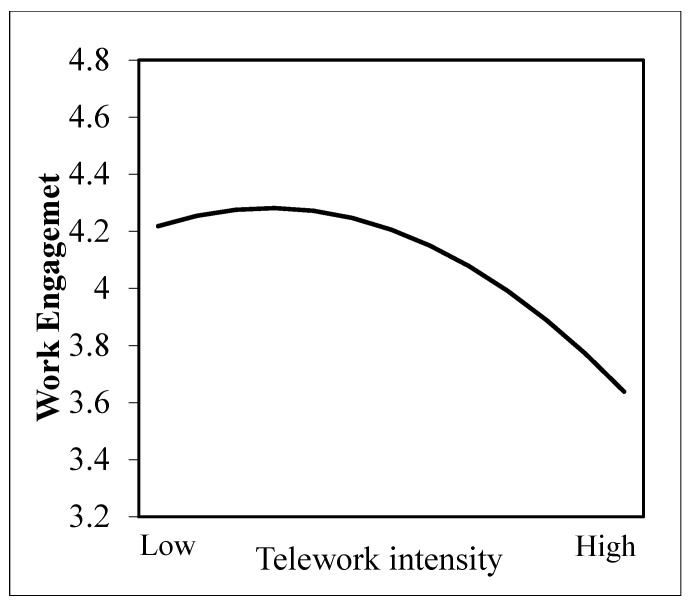
Relationship between telework intensity and work engagement.

Table 2 presents the results of the hypothesis testing. As shown in M3 (see Table 2), after removing the control variables and the main effect of telework intensity, we found a negative correlation between the squared term of telework intensity and work engagement (*b* = −0.063, *p* < 0.001). To illustrate the pattern of this curvilinear relationship, we plotted it in Figure 2 using the procedure recommended by Cohen et al. [73]. As Figure 2 displays, there is an inverted-U-shaped relationship between telework intensity and work engagement, with work engagement increasing at lower levels of telework intensity and decreasing at higher levels. Therefore, Hypothesis 1 is supported.

**Figure 3:**
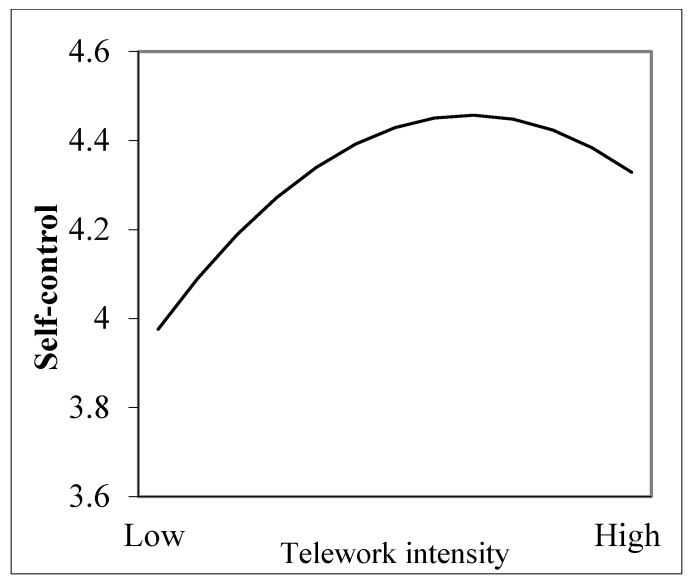
Relationship between telework intensity and self-control.

Additionally, as indicated in M1 (see Table 2), the squared term of telework intensity is negatively correlated with self-control (*b* = −0.068, *p* < 0.001). To depict the pattern of this curvilinear relationship, we plotted it in Figure 3 using procedures recommended by Cohen et al. [73]. Figure 3 shows that telework intensity had an inverted-U-shaped relationship with self-control. The relationship showed an upward trend at lower telework intensities and a downward trend at higher ones. The inflection point was calculated as 0.66 (i.e., −b_1_/(2 × b_2_) = −0.091/(2 × (−0.068)). To investigate the simple slopes of telework intensity further in predicting self-control at different intensities of telework, we calculated these simple slopes and presented them in Table 3. As shown in Table 3 (upper panel, main effect model), the simple slope is 0.27 (*p* < 0.001) when telework intensity is at its minimum value. The simple slopes become smaller in magnitude as telework intensity increases. When it reaches its maximum value, the simple slope is −0.09 (*p* < 0.05). Overall, these simple slopes show the same pattern as depicted in Figure 3, supporting Hypothesis 2.

Moreover, the results of M4 (see Table 2) indicate a positive correlation between self-control (*b* = 0.713, *p* < 0.001) and work engagement. We further conducted supplementary analyses to examine the instantaneous indirect effect. As shown in Table 4, for a low extent of telework, increasing telework intensity can function to increase work engagement through changes in self-control (as the interval estimate is entirely above zero). For a moderate extent of telework, increasing the extent can function to decrease work engagement through changes in self-control (as the interval estimate is entirely below zero). For high telework intensity, increasing the intensity would seem to have no effect on work engagement through its effect on self-control. Overall, our findings support Hypothesis 3.

**Figure 4:**
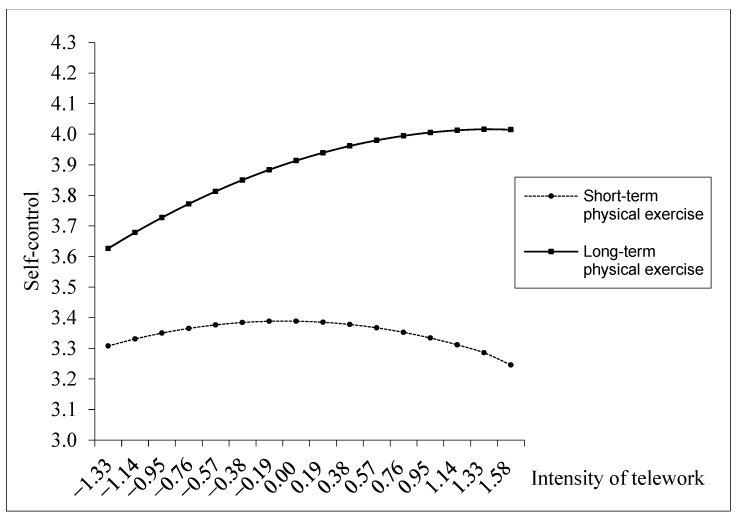
Interactive effect of telework intensity and the duration of physical exercise on self-control.

M2 (see Table 2) shows that the duration of physical exercise and telework intensity positively interact in relation to self-control (*b* = 0.049, *p* < 0.01). We plotted this moderating effect in Figure 4, showing that the inverted-U-shaped relationship between telework intensity and self-control has a higher inflection point for individuals with a higher duration of exercise, and the lateral position of the inflection point shifts to the right. Further, we calculated the conditional simple slopes and reported them in Table 3. The pattern of simple slopes for the duration of physical exercise shows that long-term physical exercise amplifies the initial positive influence of telework intensity on self-control and reduces the negative effect of high telework intensity on self-control. Significantly, long-term physical exercise delays the negative impact of telework intensity on self-control. Thus, Hypothesis 4 is supported.

## 4. Discussion

Drawing on the SRT and using data from 304 employees, we explored and tested the curvilinear relationship between telework intensity and work engagement. Additionally, incorporating the strength model of self-control, we discovered that this curvilinear relationship is mediated by self-control, with telework initially having a positive correlation with self-control. However, as telework increases, this relationship diminishes and becomes negative. Furthermore, we found that long-term physical exercise can prolong and enhance the positive impact of telework intensity on self-control, which, in turn, leads to a boost in work engagement.

### 4.1. Theoretical Contributions

Our study has made several contributions. Firstly, drawing from the SRT, we observed an inverted-U relationship between telework intensity and work engagement. This finding clarifies inconsistencies in previous research regarding the impact of telework on work engagement [8,10,11]. More importantly, our results indicate that the effectiveness of telework depends on its intensity. This discovery highlights the nuanced effects of telework intensity, revealing an optimal level of intensity that underscores the delicate balance needed in managing telework arrangements. Moreover, this insight shifts the future research focus on telework from whether to implement it to how to optimize work engagement based on the telework intensity. Our study extends the theoretical framework of the self-regulation theory (SRT) by integrating the concept of telework intensity into the analysis of work engagement. This integration offers a novel perspective on how employees’ self-regulation capacities are influenced by different levels of telework, thus advancing the theoretical understanding of employee behavior in telework.

Secondly, by integrating SRT with the strength model of self-control, we introduced self-control to elucidate the mechanism through which telework intensity affects work engagement. This mediatory pathway explores the intricate nature of how telework intensity impacts employee engagement through the lens of self-regulatory capabilities. This finding not only enriches the theoretical framework surrounding telework and employee engagement but also underscores the importance of developing self-regulation strategies to enhance work outcomes in telework. This contribution lays the groundwork for future research to explore targeted interventions that can strengthen self-control, thereby optimizing work engagement in telework. Moreover, previous research on the relationship between telework intensity and work engagement has primarily focused on external factors, such as the impact of work–family conflicts [28,29]. The analysis using self-control as a mediator offers a new internal perspective for future studies on telework’s impact on employee engagement, revealing the internal dynamics and complexity of employees under telework.

Finally, the duration of physical exercise can moderate the relationship between telework intensity and self-control, particularly where long-term physical exercise extends and amplifies the positive impact of telework intensity on self-control. This discovery represents a novel theoretical contribution, bridging the fields of physical health and work psychology. It illustrates how sustained physical activity can act as a significant buffer against the potential negative effects of high telework intensity on self-regulation. We elaborate on the notion that long-term physical exercise exerts its moderating effects through enhancing cognitive and emotional capabilities, expanding the applicability of self-regulation theory within the domain of physical health. As telework spans work and living places, our finding encourages future research to explore the interconnections between health habits and work behaviors or outcomes in telework.

### 4.2. Managerial Implications

Our findings hold significant implications in practice. Firstly, observing the inverted-U relationship between telework intensity and work engagement offers valuable insights for management. This relationship indicates an optimal level of telework intensity that maximizes work engagement. Beyond this point, increased telework intensity may have a detrimental effect on engagement, highlighting the importance of balancing telework policies. This prompts managers to reassess telework policies and practices. Organizations need to carefully adjust the amount of telework provided to employees, aiming to leverage its benefits while avoiding potential drawbacks. Implementing flexible telework policies that allow employees to adjust their work intensity could be key to maintaining high engagement levels. Additionally, providing resources and support to enhance employees’ self-regulation skills may help to maximize the benefits of telework.

Secondly, as self-control acts as a mediator between telework intensity and work engagement, it underscores the need for managers to develop strategies to enhance self-control, aiming to improve work engagement in telework. Moreover, our research confirms that high levels of self-control can enable employees to better manage their emotions and behaviors, leading to increased focus on work and higher work engagement. Therefore, during telework, managers should pay attention to the internal changes in employees and their impact on work states to more effectively mobilize their intrinsic motivation and to promote work engagement. Our study also suggests that managers should consider the individual differences in self-control among employees when designing telework policies. Tailoring telework arrangements to accommodate these differences can help to maximize work engagement across the workforce.

Ultimately, managers should encourage long-term physical exercise among employees as a strategic approach to bolster self-regulation abilities in telework. Managers should consider incorporating support for physical exercise into their telework policies, recognizing that such initiatives not only contribute to overall employee health but also enhance their capacity for effective self-management in telework. This could involve offering flexible schedules to accommodate exercise time or providing memberships for fitness programs or applications. Measures like these can help to mitigate potential declines in self-control due to high telework intensity, enabling employees to maintain their work engagement.

## 5. Conclusions and Limitations

This article investigates the curvilinear effect of telework intensity on work engagement from the perspective of the SRT, revealing that employees exhibit the highest levels of work engagement at moderate levels of telework intensity. Integrating the strength model of self-control, we discovered that self-control mediates the relationship between telework intensity and work engagement. Furthermore, the duration of physical exercise moderates this relationship, potentially extending and enhancing the positive impact of telework intensity on work engagement. Despite its theoretical and practical contributions, this study also acknowledges certain limitations. Firstly, our findings might not be universally applicable across different cultures and sectors. Future research could explore the cultural specificity of our model and its applicability in diverse organizational settings, considering the global shift towards more flexible work arrangements. Secondly, the reliance on self-reported data to validate the relationships between constructs may limit the ability to ascertain causality. Future research could benefit from incorporating objective measures, such as using objective data on physical exercise, to validate our findings. Thirdly, the study underscores that the impact of telework on work engagement depends on its intensity and frequency, suggesting that future research could examine the effects of telework intensity on other significant variables. Finally, in this study, we focused on the moderating role of the duration of physical exercise. However, the characteristics of physical exercise include its intensity, frequency, and duration [23,24]. It is undeniable that exercise frequency and intensity might also impact work engagement in telework. Future research could explore the effects of exercise frequency and intensity in telework.

## Figures and Tables

**Table 1:** Descriptive statistics and correlations.

No.	Variable	M	SD	1	2	3	4	5	6	7	8	9
1.	Gender	-	-	-								
2.	Age	-	-	0.02	-							
3.	Education	2.99	0.61	0.06	−0.05	-						
4.	Industry	4.55	3.97	−0.10	0.07	−0.09	-					
5.	Profession	2.79	1.14	−0.03	−0.09	−0.04	0.18 **	-				
6.	Telework intensity	2.49	1.33	−0.14 *	−0.07	−0.06	−0.01	0.004	-			
7.	Duration of exercise	3.10	1.58	0.01	0.02	−0.04	−0.07	−0.18 *	0.11 *	-		
8.	Self-control	3.90	0.69	0.03	0.002	−0.07	−0.14 *	−0.18 *	0.07	0.43 **	-	
9.	Work engagement	3.99	0.70	−0.02	0.08	−0.12 *	−0.13 *	−0.11	0.12 *	0.28 **	0.71 **	-

Note: *N* = 304 individuals. Gender was coded as 0 = male and 1 = female. Reliability coefficients are reported in parentheses along the diagonal. * *p* < 0.05. ** *p* < 0.01.

**Table 2:** Path modeling results.

Variables	Self-Control		Work Engagement	
	M1	M2	M3	M4
**Control variable**				
Gender	0.048	0.049	−0.012	−0.062
Age	0.011	0.005	0.061	0.048 *
Education	−0.067	−0.037	−0.124	−0.086
Industry	−0.017	−0.014	−0.020 *	−0.009
Profession	−0.090 **	−0.040	−0.049	0.014
**Predictor**				
Telework intensity	0.091 **	0.069 *	0.114 ***	
Telework intensity squared	−0.068 ***	−0.052 ***	−0.063 ***	
**Moderator**				
Duration of exercise		0.166 ***		
**Interactive terms**				
Telework intensity × Consistent physical exercise		0.049 **		
**Mediator**				
Self-control				0.713 ***
R^2^	0.103	0.257	0.082	0.518
F	4.855 ***	11.322 ***	4.883 ***	53.226 ***

Notes: *N* = 304; * *p* < 0.05, ** *p* < 0.01, *** *p* < 0.001.

**Table 3:** Simple slopes.

	Path Simple Slope (SE)		
Model	M − SD	M (0)	M + SD
Telework intensity–Self-control–Work engagement			
Main-effect model	0.27 ***	0.09 **	−0.09 *
Moderating model			
Long-term physical exercise	0.28 ***	0.15 ***	0.02
Short-term physical exercise	0.12	−0.01	−0.14 **

Notes: M − SD, M (0), and M + SD refer to the values of the mean-centering predictor variable (telework intensity). The simple slopes for a curvilinear relationship Y = b_0_ + b_1_ × X + b_2_ × X^2^ are calculated as ∂Y/∂X = b_1_ + 2 × b_2_ × X. The simple slopes for a moderating model Y = b_1_ × X + b_2_ × M + b_3_ × XM + b_4_ × X^2^ are calculated as ∂Y/∂X = b_1_ + 2 × b_4_ × X + b_3_ × M, where b_1_, b_2_, b_3_, and b_4_ are unstandardized regression coefficients. Main-effect and moderating models correspond to Table 2. * *p* < 0.05. ** *p* < 0.01. *** *p* < 0.001.

**Table 4:** Instantaneous indirect effects.

	Instantaneous Indirect Effects (95% CI)		
Model	M − SD	M (0)	M + SD
Telework intensity–Self-control–Work engagement			
Main-effect model	0.19 [0.08, 0.30]	0.06 [0.01, 0.11]	−0.06 [−0.15, 0.01]

Notes: CI refers to Monte Carlo bootstrapped confidence intervals.

## Data Availability

The data used for this study are available upon reasonable request.

## Appendix A. Measurement

**Table A1:** The scales used in this study.

Scales	Items
Telework intensity [61]	1. The average number of days a week spent working from home. The options ranged from 0.5 days to 7 days.
Self-control [62]	1. I can resist temptations very well. 2. It’s hard for me to break some bad habits. * 3. I am lazy. * 4. I say things that are inappropriate. * 5. I do things that bring me pleasure but are harmful to myself. * 6. I wish to have better self-discipline. Likert scale ranging from 1 (“strongly disagree”) to 5 (“strongly agree”).
The duration of physical exercise	1. How long can you persist in exercising (exercise such as yoga, running, aerobics, cycling, etc.). Options included 0–3 months, 3–6 months, 6–12 months, 1–2 years, and two years and above.
Work engagement [63]	1. At work, I feel energetic.
	2. At work, I feel strong and vibrant.
	3. In the morning, I am eager to go to work.
	4. I am passionate about my job.
	5. My work inspires me.
	6. I am proud of the work I do.
	7. I feel happy while working.
	8. I am fully immersed in my work.
	9. While working, I concentrate on the task at hand and forget about other things. Likert scale ranging from 1 (“strongly disagree”) to 5 (“strongly agree”).

Note: * Item is reverse-scored.
